# Indoor damp surfaces harbor molds with clinical significance

**DOI:** 10.18502/cmm.4.3.169

**Published:** 2018-09

**Authors:** Azadeh Habibi, Banafsheh Safaiefarahani

**Affiliations:** 1 Department of Biodiversity, Institute of Science and High Technology and Environmental Sciences, Graduate University of Advanced Technology, Kerman, Iran; 2 Plant Protection Research Department, Fars Agricultural and Natural Resources Research and Education Center, AREEO, Shiraz, Iran

**Keywords:** Fungal diversity, Molecular identification, Mycoses

## Abstract

**Background and Purpose:**

Fungal contamination in damp places in buildings has become an increasing problem worldwide. Dampness facilitates the growth of fungi, which can cause adverse effects not only on the buildings but also on their occupants. The aim of this study was to identify indoor mold species in the buildings of Kerman province, Iran.

**Materials and Methods:**

In this study, 110 samples were obtained from surfaces of damp indoor areas in buildings randomly selected in Kerman province. The identification of fungal species was based on the macroscopic and microscopic characteristics of the isolates, such as colony morphology, hyphae, conidia, and conidiophores, as well as molecular sequence data.

**Results:**

Based on the results, a total of 218 fungal isolates were obtained. Apart from frequently isolated fungi, such as *Alternaria*, *Aspergillus*, and *Penicillium*, 13 species, including *Cladosporium sphaerospermum*, *Cladosporium herbarum*, *Cladosporium halotolerans*, *Engyodontium album*, *Collariella bostrychodes*, *Stachybotrys xigazenensis*,* Ramularia eucalypti*, *Fusarium merismoides*, *Fusarium solani*, *Ochroconis musae*, *Mucor racemosus*, *Acremonium zonatum,* and *Acremonium persicinum* were identified, and the selected species were described. Among these 13 species, *Cladosporium *was the most common species (43%) in indoor surfaces, followed by *Ochroconis musae* (10.8%) and *Engyodontium album *(7.4%). To the best of our knowledge, *Stachybotrys xigazenensis* was reported in the present study for the first time in Iran. In addition, *E. album* and *O. musae* were isolated for the first time from indoor surfaces in Iran.

**Conclusion:**

According to the results, the level of overall fungal richness across indoor surfaces was high. Some of the isolated taxa were clinically significant. It was concluded that the damp residential surfaces were potentially passive collectors of clinically significant molds.

## Introduction

In daily life, people are exposed to thousands of mold species in buildings some of which are known human pathogens or serve as the triggers of allergies and allergenic asthma. The exposure of people to various indoor microbial communities has been increasing considering the amount of time spent indoors due to the global trends towards industrialization and urbanization [1-3]. Fungi may cause allergic mycosis, hypersensitivity pneumonitis, and fungal sinusitis in human [4]. The indoor mold growth in damp buildings has been shown to be associated with an increase in allergy and symptoms of the sick building syndrome [5, 6]. 

Fungi may colonize human body or cause damage by the production of toxins, enzymes [7], and volatile organic compounds [8], such as certain ketones (e.g., 2-heptanone), alcohols (e.g., 1-octen-3-ol), terpenes and terpene derivatives (e.g., geosmin), and sulfur compounds (e.g., dimethyl disulfide) [6, 9, 10]. Children living in buildings with high levels of microbial volatile compounds have shown more symptoms of asthma, hay fever, wheezing, and eye irritation [11]. Depression is proved to be associated with indoor molds in Europe [12].

The indoor mold growth is not a new problem. In this regard, there is a report on the production of a poison gas and illness in occupants resulting from *Scopulariopsis brevicaulis* (Sacc.) Bainier growth in buildings in the nineteenth century [13]. Several researchers have addressed fungi in indoor environments [14-25]. In Iran, Hedayati *et al*. [24] showed the high concentration levels of common allergenic molds, namely *Cladosporium*, *Aspergillus*, *Penicillium*, and *Alternaria,* in indoor areas of the houses of patients suffering from asthma. Foladi *et al*. [25] reported *Cladosporium* spp., *Aspergillus* spp., *Penicillium* spp., and *Stachybotrys* *chartarum* as the most frequent taxa isolated from the surface samples in the archives of different offices in Sari, Iran.

Fungi are able to inhabit diverse niches wherever there is moisture and a food source particularly in damp or water-damaged places. Since the diversity and concentration of indoor molds are important factors interacting with occupants’ health, it is critical to understand the taxonomic composition of fungal species in indoor spaces. This study was conducted to identify indoor fungal species and monitor their prevalence in the buildings of Kerman, Iran, for the first time. 

## Materials and Methods

*Isolation of indoor fungi*

In 2017, a total of 110 samples were obtained from damp indoor places of the buildings randomly selected in Kerman, the second largest province of Iran with a land area of 180,726 km^2^ that encompasses nearly 11% of this country. The selection of damp places was based on the visual inspection of the buildings for the presence of visible mold growth, water damage, water leakage, humidification, defective plumbing instructions, and condensation. Each building was sampled once and at five different sites using a swab for surfaces (*i.e*., walls and floor). 

Air sampling methods were excluded to avoid bias in favoring fungal isolates with large quantities of small dry spores. In addition, public buildings, including hospitals, were excluded from the present study. Malt Extract Agar (MEA) and Potato Dextrose Agar (PDA) supplemented with antibiotics were used for fungal isolation. Pure cultures were established using single spore or hyphal tip techniques. All the isolates were deposited in Fungal Culture Collection in Kerman Graduate University of Advanced Technology, Kerman, Iran. 

*Morphological identification of isolates*

The identification of fungal species was based on the macroscopic and microscopic characteristics of the isolates, such as colony morphology, hyphae, conidia, and conidiophores. Colony morphology was assessed on the PDA after 7-14 days at 25°C. Lactophenol, lactophenol cotton blue, and 50% lactic acid solutions were used to prepare microscopic slides. The measurement and microphotographs of fungal features were taken from agar plates and microscopic slides using Dino-eye microscope camera USB lens (The Microscope Store, LLC., USA). A total of 30 measurements were obtained from the relevant parameters of conidiophores and conidia.

*Molecular examination of isolates*

The molecular identification of the fungal isolates was confirmed by DNA sequence analysis. To this end, the isolates were grown on PDA for 7-15 days at 25˚C. Approximately 100 mg of fresh mycelia was scraped off the PDA plate of each isolate and homogenized using liquid nitrogen. The cells were lysed using cetyl trimethylammonium bromide solution, and the DNA was extracted using DNG^TM^-Plus solution (Sinaclon, Iran) following the manufacturer’s instructions [26]. The DNA concentrations were estimated by a NanoDrop spectrophotometer (NanoDrop Technologies, USA). 

The ITS1-5.8S-ITS2 rDNA regions were amplified using two primers ITS1 and ITS4 [27]. A 700 bp portion of the translation elongation factor 1ɑ (*tef1-alpha*) gene was amplified from *Fusarium* isolates using the primers EF1 (5'-ATG GG TAA GGA RGA CAA GAC-3') and EF2 (5' GGA RGT ACC AGT SAT CAT G-3') [28]. Primer pairs, namely ACT-512F and ACT-783R [29], were utilized to amplify a partial fragment of the actin gene in *Cladosporium* spp. Twenty-five μL polymerase chain reaction (PCR) contained 1X reaction buffer, 0.4 mM of each primer, 200 mM dNTPs, 2.5 mM MgCl_2_, 20 ng of DNA, and 1 unit of Taq polymerase. 

The PCRs were performed in a Biometra TAdvanced Thermal Cycler (Biometra, Göttingen, Germany). The cycling conditions consisted of 94°C for 3 min, followed by 30 cycles of 94°C for 30 sec, 60°C for 30 sec, and 72°C for 1 min, and then 5 min at 72°C for ITS1 and ITS 4. The annealing temperature of 54°C was used for *tef1-alpha*. The sequencing was carried out by Macrogen (Macrogen Inc., South Korea). The sequences were manually edited by Geneious Prime (Biomatters Inc., USA). The results of sequenceing were compared with the findings in NCBI/Genbank database using a basic local alignment search tool [30]. 

## Results

*Fungal isolates*

**Figure 1:**
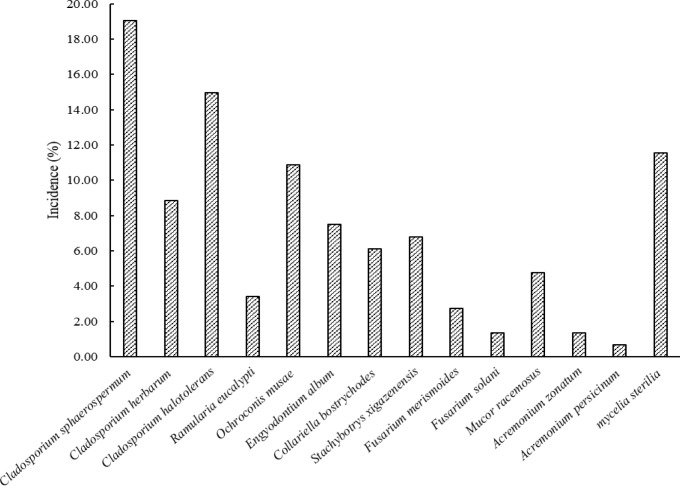
Incidence rate of indoor fungal species in the damp environments of buildings in Kerman, Iran

In this study, a total of 218 fungal isolates were obtained. The sequences generated in this study were deposited in GenBank under the accession numbers MH931817 to MH931829 and MH933701 to MH933703. The morphological and molecular investigations led to the identification of 13 species. These species included *Cladosporium sphaerospermum*, *Cladosporium herbarum*, *Cladosporium halotolerans*, *Engyodontium album*, *Collariella bostrychodes*, *Stachybotrys xigazenensis*,* Ramularia eucalypti*, *Fusarium merismoides*, *Fusarium solani*, *Ochroconis musae*, *Mucor racemosus*, *Acremonium zonatum*, and *Acremonium persicinum*. Figure 1 demonstrates the isolation frequency of the identified species. 

*Taxonomy*

***Cladosporium sphaerospermum***

**Figure 2:**
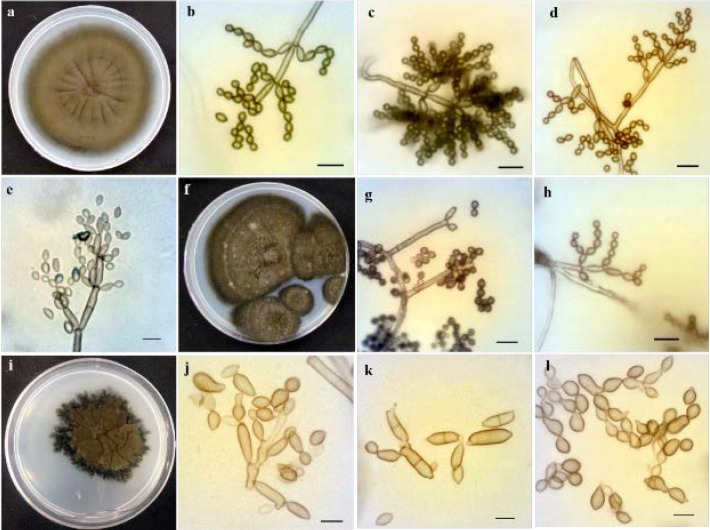
*Cladosporium sphaerospermum*: a) colony incubated for 14 days at 25°C grown on Potato Dextrose Agar (PDA), b-d) conidiophores and conidia, e) ramoconidia and conidia; *Cladosporium halotolerans*: f) colony incubated for 16 days at 25°C grown on PDA, g) conidiophores with denticles, h) conidia; *Cladosporium herbarum*: i) colony incubated for 21 days at 25°C grown on PDA, j) nodulose conidiogenous cells with sympodial proliferation, k) ramoconidia, l) conidia with distal hila (scale bars=10 µm)

Figure 2a-e illustrates* Cladosporium sphaerospermum* Penz, Michelia 2 (8): 473 (1882). *Description:* Colonies on PDA reaching 20-40 mm in diameter during 14 days at 24°C, grey olivaceous, margin white, regular. Conidiophores erect, arising terminally and laterally from hyphae, 40-130×2.5–4.3 μm. Conidiogenous cells have single or few apical scars and are terminal (sometimes intercalary), 6-15 μm long, with sympodial proliferation. Conidia produced in branched chains, aseptate, globose to subglobose or ovoid; sometimes, both end narrower, 2-5×2.5-3.5 μm. Ramoconidia cylindrical, sometimes ellipsoid, 0-3 septation. 

***Cladosporium herbarum***

Figure 2i-l depicts *Cladosporium herbarum* (Pers.) Link, Magazin der Gesellschaft Naturforschenden Freunde Berlin 8: 37 (1816). 

*Description:* Colonies on PDA reaching 15-30 mm in diameter during 14 days at 24°C, olivaceous to dark olivaceous, fluffy, margins almost black, irregular, aerial mycelium abundant and dense, sporulation abundant, radially furrowed in older cultures, folded and wrinkled in the colony center, without prominent exudates. Conidiophores erect, straight to flexuous, nodulose, with multilateral swellings giving the stalk a knotty appearance, pale brown, smooth, walls thickened. Conidiogenous cells nodulose with 1-5 swellings per cell, proliferation sympodial, protuberant. Conidia produced in unbranched chains, aseptate, intercalary conidia with 1-3 distal hila, ellipsoid to cylindrical, 6-12×3.5-5 μm. Ramoconidia cylindrical, sometimes ellipsoid, 0-3 septation.

***Cladosporium halotolerans***

Figure 2f-h displays* Cladosporium halotolerans* Zalar, de Hoog, and Gunde-Cimerman, Studies in Mycology 58: 172 (2007).

*Description:* Colonies on PDA reaching 30-45 mm in diameter during 14 days at 24°C, dark olive, showing some furrows with age, reverse black. Conidiophores erect, mostly smooth, and unbranched, having obvious denticles, walls thin. Conidia produced in branched chains, brown, aseptate, globose to subglobose or few ovoid ones, sometimes narrower at both ends, 2-5×2.5-3 μm. Ramoconidia barely observed, cylindrical to spherical, 0-1 septation. Conidiogenous cells protuberant. 

***Ramularia eucalypti***

**Figure 3:**
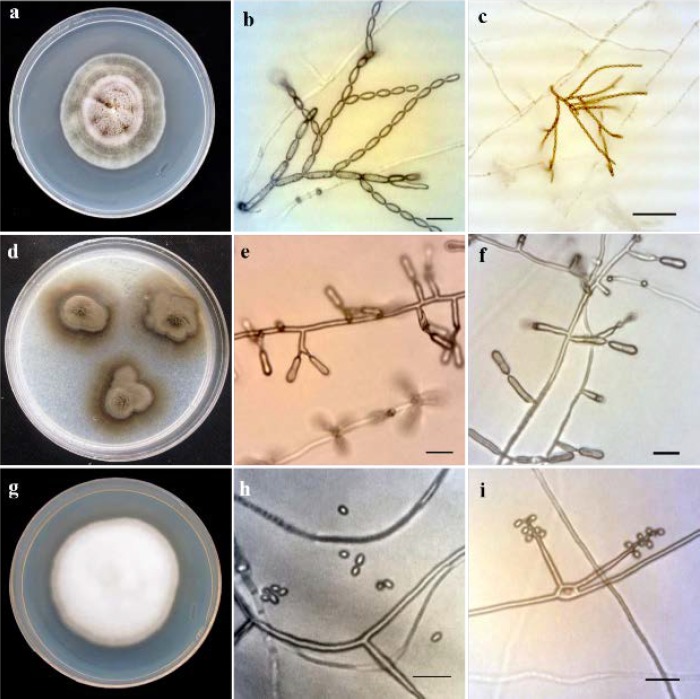
*Ramularia eucalypti*: a) colony incubated for 10 days at 25°C grown on Potato Dextrose Agar (PDA), b-c) conidiophores and conidia; *Ochroconis musae*: d) colony incubated for 14 days at 25°C grown on PDA, e-f) conidiophores and conidia; *Engyodontium album*: g) colony incubated for 7 days at 25°C grown on PDA, h) conidia, i) conidiophores and conidia (scale bars=10 µm, c=50 µm)

Figure 3a-c presents* Ramularia eucalypti* Crous, Fungal Diversity 26: 174 (2007). 

*Description*: Colonies on PDA reaching 30-40 mm in diameter in 21 days at 24°C, erumpent, convex, radially striated, dirty white with pale grey olivaceous tones, Conidiogenous cells hyaline, smooth, 9-20×2-3 μm, with 1-2 apical loci. Ramiconidia hyaline, smooth, aseptate, 5-12×2-3 μm, conidia hyaline, smooth, fusiform, 4-6×1.5-3 μm. 

***Ochroconis musae***

Figure 3d-f depicts *Ochroconis musae* (G.Y. Sun and Lu Hao) Samerpitak and de Hoog, Mycological Progress 14 (2/6): 8 (2015). 

*Description*: Colonies on PDA reaching 30-35 mm in diameter during 21 days at 24°C, brown to dark brown, smooth, with prominent brown exudates. Conidiophores mostly cylindrical to needle-shaped, bearing a few conidia near the apex. Conidia two-celled, smooth-walled, subhyaline to pale brown, cylindrical, 9-12.5×4-6 μm. 

***Engyodontium album***

Figure 3g-i illustrates* Engyodontium album* (L imber) de Hoog, Persoonia 10 (1): 53 (1978).

*Description:* Colonies on PDA reaching 30-40 mm in diameter during 7 days at 24°C, white, cobweb to floccose and reverse side uncolored. Conidiophores hyaline and thin-walled. Conidiogenous cells forming in whorls terminating in a zigzag rachis bearing hyaline, smooth walled, one celled, globose conidia on denticles.

**Figure 4:**
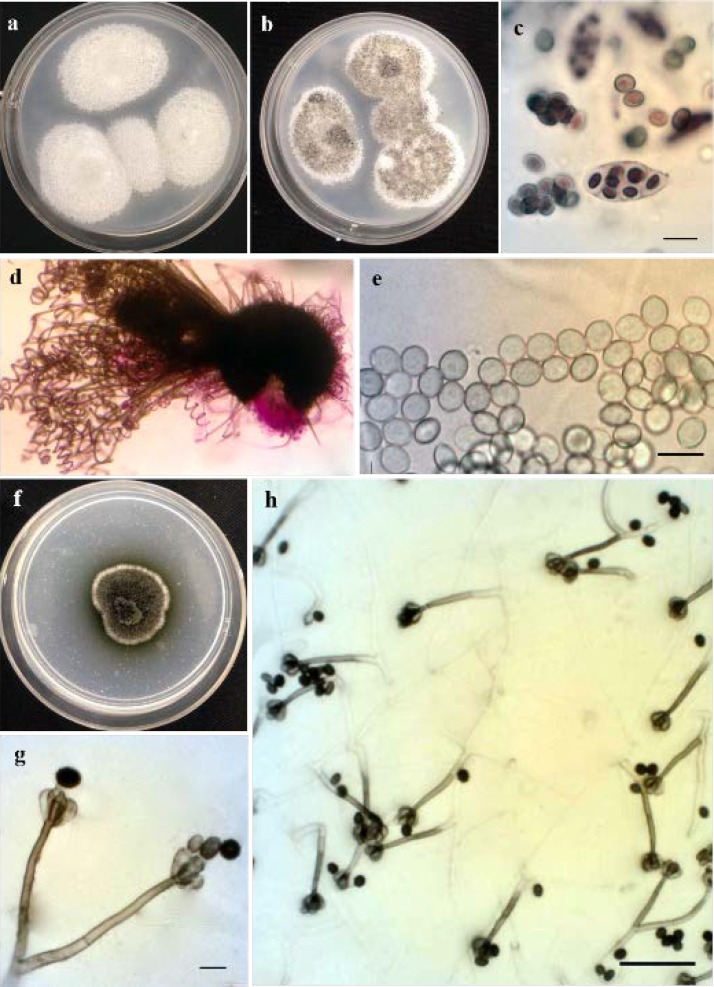
*Collariella bostrychodes*: a-b) colony incubated for 14 days and 25 days at 25°C grown on Potato Dextrose Agar (PDA), c) asci, d) ascomata mounted in lactophenol acid fuchsin, e) ascospores; *Stachybotrys xigazenensis*: f) colony incubated for 7 days at 25°C grown on PDA, g-h) conidiophores, conidia, and conidiogenous cells (scale bars=10 µm, h=50 µm)

***Collariella bostrychodes***

Figure 4a-e shows *Collariella bostrychodes* (Zopf) X. Wei Wang and Samson, Studies in Mycology 84: 179 (2016).

*Description:* Colonies on PDA reaching 30-40 mm diameter during 7 days at 24°C, aerial hyphae sparse, white; reverse side of colony uncolored. Ascomata green to greyish, superficial, subglobose to ovate, ostiolate, 165-250 μm in diameter. Ascomatal hair 4-7 μm in diameter at base, dark brown, septate, straight in the lower part, spirally coiled in the upper part. Asci fasciculate, clavate, eight-spored, 20-33×7-12 μm. Ascospores limoniform, 6.5±0.3×5.9±0.1×5.2±0.2 μm, n=30.

***Stachybotrys xigazenensis***

Figure 4f-h depicts* Stachybotrys xigazenensis* Y.M. Wu and T.Y. Zhang, Mycotaxon 114: 461 (2011).

*Description:* See Wu and Zhang [31]. This fungus differs from *S. chartarum* in conidial size, the conidia of *S. xigazensis* were wider than those of *S. chartarum* (7-12×4-6 µm). 

**Figure 5:**
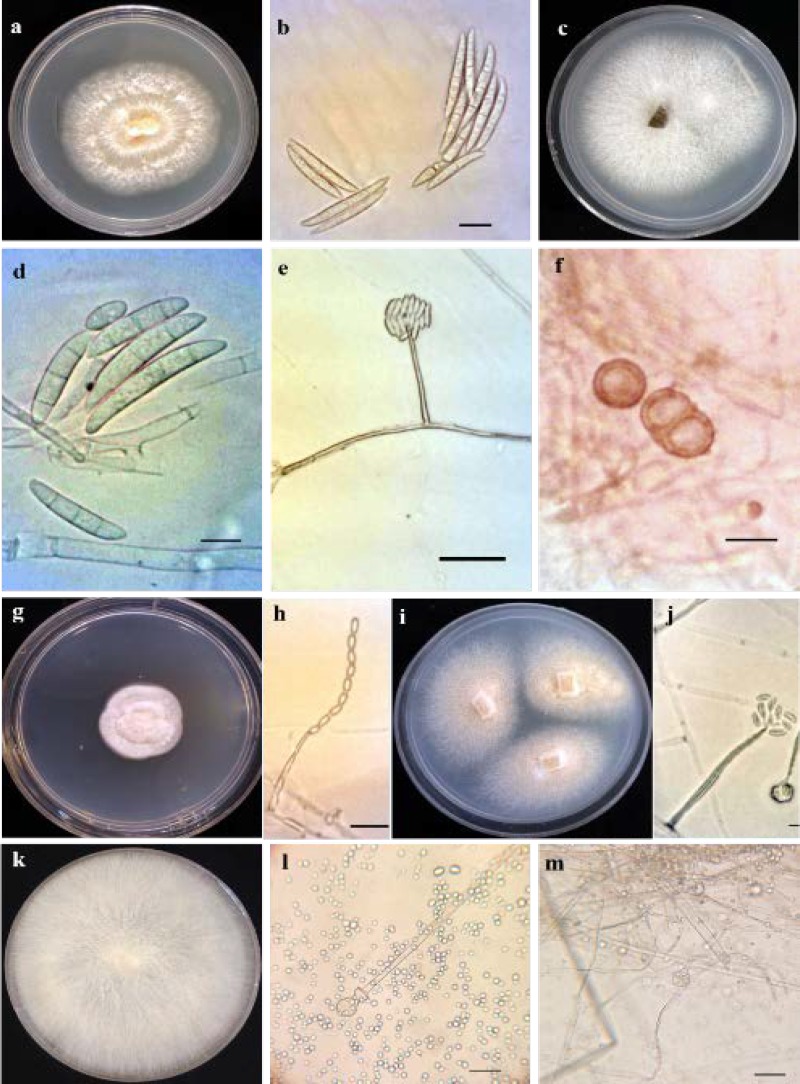
*Fusarium merismoides*: a) colony incubated for 60 days at 25°C grown on Potato Dextrose Agar (PDA), b) macroconidia; *Fusarium solani*: c) colony incubated for 5 days at 25°C grown on PDA, d) macroconidia, e) false head, f) chlamidospore; *Acremonium persicinum*: g) colony incubated for 10 days at 25°C grown on PDA, h) phialid and spores; *Acremonium zonatum*: i) colony incubated for 10 days at 25°C grown on PDA, j) phialid and spores; *Mucor racemosus*: k) colony incubated for 5 days at 25°C grown on PDA, l-m) sporangium and sporangiospores (scale bars=10 µm, e=50 µm)

Colony morphology and microscopic characteristics of *Fusarium merismoides*, *F. solani*, *Mucor racemosus*, *Acremonium persicinum*, and *Acremonium zonatum* are shown in Figure 5. 

## Discussion

The results of the present study revealed that a variety of fungal species constituted the indoor fungal contaminations of the buildings in Kerman, Iran. The frequently isolated fungi, such as *Alternaria*, *Aspergillus*, and *Penicillium,* had the most predominant species representing 32.5% (n=71) of all the isolates, which is consistent with other relevant studies [32-35]. Up to now, most of the studies regarding indoor fungal flora have focused on frequently isolated taxa, such as the species of *Aspergillus*, *Alternaria*, and *Penicillium* [14-23]. 

The importance of less frequently isolated but clinically significant fungal species may have been underestimated. Therefore, the species of *Alternaria*, *Aspergillus*, and *Penicillium* were not precisely considered in this study to avoid duplicates and overlap with most of the indoor molds studies. The present study involved no evidence on the molds causing allergies or diseases.

In the present study, *Cladosporium* was the dominant (42.8%) genus in concordance with the results of several other studies [36-43]. Accordingly, *C. sphaerospermum* (19%) was found as the dominant species, followed by *C. halotolerans* (15%) and *C. herbarum* (8.8%). The members of *C. sphaerospermum* dealt with low-nutrient conditions and lowered water activity, compared to other species [39], which is consistent with Kerman natural environment. The *Cladosporium* species reported in this study may represent a threat to individuals since they are described to cause fungal allergies, especially in patients with severe asthma [42, 44] and may need further investigations. 

*Stachybotrys xigazenensis* was reported in this study for the first time in Iran. Without the careful examination of conidia, this species can be confused with *Stachybotrys chartarum*. This fungus differs from *S. chartarum* in conidial size; in this regard, the conidia of *S. xigazensis* are wider than those of *S. chartarum*. *S. chartarum* is strongly cellulolytic and is reported as one of the causes of the sick building syndrome [45, 46]. This species causes mycotoxicosis via the production of mycotoxins, such as trichothecenes, and is referred to as the black toxic mold [47]. The ecological and clinical signiﬁcance of *S. xigazensis* need further investigations regarding indoor environments in Kerman. 

This is the first study reporting the presence of *Ochroconis musae* (*O. mirabilis*) in indoor surfaces as one of the indoor microbiota. It was revealed that* Ochroconis musae* had clinical importance causing mild cutaneous infections in humans [48-51]. Lian and de Hoog [52] discussed the possibility of some strains entrance to the softened human skin during bathing. The clinical significance of these isolated strains remains enigmatic and needs to be investigated in future studies. 

*Engyodontium album* is a keratinophilic fungus and has been reported to cause keratitis [53], brain abscess [54], fungaemia, eczema vesiculosum cerebritis, and endocarditis [55, 56]. Kachuei et al. [57] isolated *E. album* from the soil in Isfahan province, Iran. In the present study, *E. album* was isolated for the first time from moist surfaces in water-damaged buildings in Kerman. *E. album* has a halophilic nature; therefore, future studies can examine the correlation between the prevalence of this species and water properties in Kerman. 

*E. album* used to be included in the genus *Beauveria* described by Vuillemin [58]. Subsequently, Limber [59] categorized it in a new genus, namely *Tritirachium*. De Hoog [60] described a new genus, called *Engyodontium*, which consists of two species, namely *E. album* and *E. parvisporum*. This species differs from *Tritirachium* species regarding the lack of pigmentation and from *Beauveria* species considering the presence of conidiogenous cells in whorls [53].

*Mucor racemosus*, *Collariella bostrychodes* (formerly known as *Chaetomium bostrychodes* Zopf), *F. merismoides*, *F. solani*, *R. eucalypti,* *A. zonatum,* and *A. persicinum* were also isolated with the frequency rates of 4.7%, 6.1%, 2.7%, 1.3%, 3.4%, 1.3%, and 0.6%, respectively. These species have a clinical significance and have been reported to cause a broad spectrum of human diseases [40, 61-64, 13]. 

## Conclusion

The present study presented fungal species obtained from buildings, especially damp places, along with their clinical significance in humans. Based on the findings, it can be concluded that damp residential surfaces are collectors of clinically significant molds and involve in the growth, distribution, and transmission of these species. The present research can be regarded as a step forward in understanding the taxonomic composition of clinically important fungal species in buildings. These findings suggest that occupants have to ask questions about the probable hazards of the molds growing in damp places in their buildings, which require further investigations.
